# Pulmonary venous occlusion and death in pulmonary arterial hypertension: survival analyses using radiographic surrogates

**DOI:** 10.1186/1471-2466-11-47

**Published:** 2011-10-06

**Authors:** Yasuko Takeda, Yutaka Takeda, Koji Yamamoto, Shigehiro Tomimoto, Tomomitsu Tani, Hitomi Narita, Nobuyuki Ohte, Genjiro Kimura

**Affiliations:** 1 Department of Cardio-Renal Medicine and Hypertension, Nagoya City University Graduate School of Medical Sciences, Nagoya, Japan; 2 Department of Cardiology, Nagoya City Rehabilitation Center, Nagoya, Japan

## Abstract

**Background:**

Recent studies find that a considerable number of patients with pulmonary arterial hypertension (PAH) develop fibrous obstruction of the pulmonary veins. Such obstruction more commonly accompanies connective tissue disorder (CTD)-associated PAH than idiopathic PAH. However, few researchers have gauged the risk of death involving obstruction of the pulmonary veins.

**Methods:**

Thirty-seven patients with PAH were enrolled (18 patients, idiopathic PAH; 19 patients, CTD-associated PAH). The patients were 49 ± 18 years and had a World Health Organization functional class of 3.2 ± 0.6. Thickening of the interlobular septa, centrilobular ground-glass attenuation, and mediastinal adenopathy were surrogates for obstruction of the pulmonary veins, and were detected by a 16-row multidetector computed tomography scanner.

**Results:**

The follow-up period was 714 ± 552 days. Fifteen deaths occurred. Thickening of the interlobular septa, centrilobular ground-glass attenuation, and mediastinal adenopathy were found in 37.8%, 24.3%, and 16.2% of patients, respectively. Cox proportional hazard analysis revealed an increased risk of death with each radiographic surrogate (mediastinal adenopathy: *p *< 0.0001, hazard ratio = 13.9; thickening of interlobular septa: *p *< 0.001, hazard ratio = 12.0; ground-glass attenuation: *p *= 0.02, hazard ratio = 3.7). The statistical significance of these relationships was independent of the cause of PAH and plasma concentration of brain natriuretic peptide.

**Conclusions:**

The results of this study imply that obstruction of the pulmonary veins is associated with an increased risk of death in patients with PAH.

## Background

Pulmonary arterial hypertension (PAH) carries a significant risk of death [1,2]. Patients with a poor response to pulmonary vasodilating agents often die within several years [1]. Recent studies show that a considerable number of patients with PAH develop fibrous obstruction in the pulmonary veins [3,4]. These findings challenge relationship between clinical entity of PAH and that of pulmonary veno-occlusive disease (PVOD). Case reports suggest that obstruction of pulmonary veins is associated with a poor prognosis [5-8]. However, few researchers have gauged the risk of death due to pulmonary venous obstruction. To assess the effect of pulmonary venous obstruction on the risk of death in patients with PAH, this study performed survival analyses with patients grouped by the cause of PAH and by the presence or absence of radiographic surrogates for obstruction of the pulmonary veins.

## Methods

### The patients

Forty-five patients with idiopathic PAH or connective tissue disorder (CTD)-associated PAH were referred to our PAH clinic from January 2004 to March 2009. We excluded eight patients from analyses for the following reasons: a total lung capacity less than 70% of the predicted value (two patients); a serum creatinine concentration greater than 2.5 mg/dL (two patients); an age greater than 75 years (two patients); the presence of liver cirrhosis (one patient); and the presence of gastric cancer (one patient). The remaining 37 study participants (8 men and 29 women) gave informed consent. PAH was diagnosed from the results of right heart catheterisation at rest [9,10]. PAH was defined as a mean pulmonary artery pressure greater than 25 mm Hg, a pulmonary capillary wedge pressure less than or equal to 15 mm Hg, and pulmonary vascular resistance greater than 3 Wood units. All of the patients completed the evaluations, as described below. However, the data of right heart catheterisation from 11 patients were not used for analyses for the change of the treatment between catheterisation and the other baseline evaluations. Pulmonary arterial thromboembolism was not detected in any of the patients with a lung perfusion scan. The institutional ethics committee approved the protocol. All the participants gave informed consent to this study.

### Assessment of radiographic surrogates of pulmonary venous obstruction

**Figure 1:**
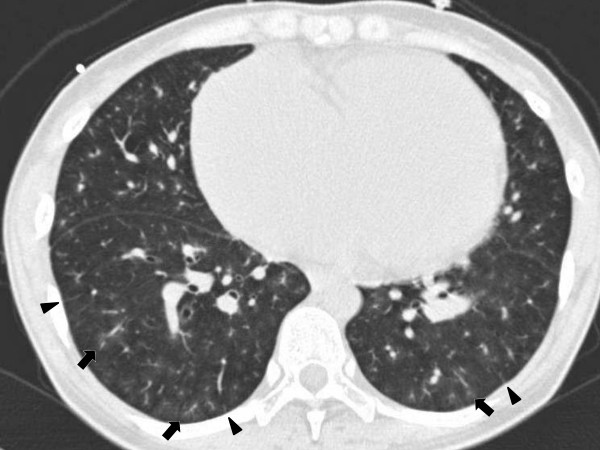
**The computed tomography scan of a 25-year-old woman with severe idiopathic pulmonary arterial hypertension reveals thickening of the interlobular septa (arrowheads) and small multifocal areas of centrilobular ground-glass attenuation**.

Thickening of the interlobular septa, the presence of centrilobular ground-glass attenuation, and enlargement of the mediastinal lymph nodes were surrogates for obstruction of the pulmonary veins; these surrogates were detected with computed tomography (CT) scans (Figure 1) [5,7-9,11-16]. The details of these abnormalities are described elsewhere [15,17-19]. A 16-row multidetector CT scanner (IDT 16, Philips Electronic Japan Medical Systems, Tokyo, Japan) used the following scan parameters: simultaneous acquisition of 16 slices per rotation with a slice thickness of 0.75 mm, rotation time of 500 ms, pitch of 0.9 mm, tube voltage of 120 kV, and tube current of 200 mA. Cross-sectional images were reconstructed with a slice thickness of 3.0 mm at 3.0-mm intervals. Board-certified radiologists gave reports on the CT images within 24 hours after the scans. Our radiologists were not restricted from other clinical data. Two investigators assessed the CT images and the reports for the surrogates within a few days thereafter. Final decisions were reached by consensus if there was an inconsistency.

### Assessment of the pulmonary hypertension status

One investigator assessed each patient's functional class in accordance with the World Health Organisation (WHO) criteria. The patients underwent an overnight fast. Blood samples were afterwards drawn from a peripheral vein. The samples were immediately placed on ice and centrifuged at 4°C. The plasma concentration of brain natriuretic peptide (BNP) was then measured. An external laboratory (SRL Inc., Tokyo, Japan) measured BNP concentrations using an immunoradiometric assay. A trained sonographer recorded all echocardiograms using a commercially available system (SSA-770A, Toshiba Medical Co. Ltd, Tokyo, Japan). The cardiac index and Doppler right ventricular index were calculated using Doppler echo techniques [20]. Each measurement was the average of three or more consecutive beats at the end expiration.

### Follow-up

The baseline was defined as the day of the CT scan. CT scan was taken just before initiation of treatment in out institute. The patients were asked to visit our outpatient clinic every four weeks to adjust medical therapy and to obtain information on clinical events. In addition to these periodical contacts, the investigators and the patients sometimes communicated by phone. (If a patient did not come, the investigators interviewed the patients or their family by phone.) The treatment was not controlled because of the generally high mortality. Treatment failure was defined as death, hospitalisation due to cardiovascular events (including syncope), use of inotropic agents, elevation of a patient's WHO functional class, or a three-fold increase in the plasma BNP concentration [21].

### Statistical analyses

Statistical analyses were performed with the Statistical Package for Social Science version 15.0 for Windows (SPSS Inc., Chicago, Illinois). Survival times were defined as the length of time a patient lived from the baseline date to Dec 31, 2009. Data are expressed as the mean ± the standard deviation. The plasma BNP concentrations were transformed to their natural logarithms to normalise the distribution, and the BNP data are expressed as the median with the 25th and 75th percentiles. Categorical variables were compared using the *chi*-square test. The effect of each radiographic surrogate for pulmonary venous obstruction and the effect of each cause of PAH in relation to the patients' characteristics were tested with a two-way analysis of variance. The prognostic values of the variables were tested using Cox proportional-hazards regression analyses. The results are expressed as the hazard ratios with 95% confidence intervals. The Kaplan-Meier method produced the survival curves. A two-sided *p *value less than 0.05 was considered statistically significant.

## Results

### Patients' characteristics and follow-up results

Table 1 shows the characteristics of the patients. The follow-up period was 714 ± 552 days. All patients maintained their follow-up visits. Death occurred in 5 (24%) class III patients and in 10 (83%) class IV patients, but no deaths occurred in class II patients (*p *< 0.001). Death occurred in 9 (50%) in patients with idiopathic PAH and in 6 (32%) in patients with CTD-associated PAH (p = 0.23). Heart failure was the cause of death in all but two mortality cases. One of these two patients died of septic shock and the other died of a massive lung haemorrhage. Treatment failure occurred in 10 (83%) class IV patients; nine (43%) class III patients; and one (20%) class II patient (*p *= 0.038). No patient received lung or heart-lung transplantation. All patients experienced an increase in the use of pulmonary vasodilating agents.

**Table 1:** Baseline characteristics of the patients

Variables	n = 37
Age (yrs)	49.0 ± 18.1
Women	29 (78.4)
Time from the onset to study enrolment (weeks)	14.2 ± 18.3
Functional class II/III/IV	4/21/12 (10.8/56.8/32.4)
Idiopathic PAH/connective tissue disorder-associated PAH	18/19 (48.6/51.4)
Death	15 (40.5)
Treatment failure*	20 (54.1)
Use of PAH-specific drug at baseline	
Epoprostenol alone	11 (29.7)
Sildenafil alone	4 (10.8)
Bosentan alone	6 (16.2)
Beraprost alone	13 (35.1)
Epoprostenol + Sildenafil	1 (2.7)
Sildenafil + Beraprost	1 (2.7)
Sildenafil + Bosentan + Beraprost	1 (2.7)
Oxygen	33 (89.2)

Values are presented as the number (percent) or as the mean ± standard deviation (SD).

*Treatment failure was defined as death, hospitalisation due to cardiovascular event (including syncope), use of inotropic agents, or elevation in a patient's WHO functional class.

Abbreviation: PAH, pulmonary arterial hypertension.

### Radiographic surrogates for obstruction of the pulmonary veins

The frequency of each radiographic surrogate for pulmonary venous obstruction was shown in Table 2. There was no statistical difference between the causes of PAH in the frequency of each surrogate (mediastinal adenopathy: *p *= 0.21; thickening of interlobular septa: *p *= 0.42; ground-glass attenuation: *p *= 0.77). The haemodynamic data and plasma concentration of BNP were comparable between the causes of PAH. Mediastinal adenopathy was associated with an advanced age and a high plasma concentration of BNP. Thickening of interlobular septa was associated with a low cardiac index and high plasma concentration of BNP. Centrilobular ground-glass attenuation was not associated with any of the haemodynamic data or with the plasma concentration of BNP.

**Table 2:** Characteristics of the patients with each sign of pulmonary venous obstruction

Signs		Cause	n	Age(years)	**mRAP(mm Hg)**^**§**^	**mPAP (mm Hg)**^**§**^	CI(L/min/m2)	BNP(pg/mL)
Mediastinal	Yes	Idiopathic	6	56 ± 18*,^#^	9 ± 4	43 ± 5	1.9 ± 0.4	860 [637 - 1210]**
		
adenopathy		CTD	3	65 ± 3*,^#^	8 ± 3	36 ± 19	2.5 ± 0.3	148 [83 - 959]**
	
	No	Idiopathic	12	37 ± 17*,^#^	8 ± 3	62 ± 16	2.4 ± 0.7	133 [37 - 418]**
		
		CTD	16	52 ± 16*,^#^	7 ± 4	48 ± 19	2.5 ± 0.9	141 [75 - 334]**

Thickening of	Yes	Idiopathic	8	50 ± 19	9 ± 5	56 ± 16	1.9 ± 0.4*	800 [487-1062]*
		
interlobular septa		CTD	6	49 ± 18	7 ± 4	42 ± 18	2.0 ± 0.4*	482 [83-959]*
	
	No	Idiopathic	10	38 ± 19	7 ± 2	56 ± 16	2.5 ± 0.7*	92 [28 - 333] *
		
		CTD	13	56 ± 14	7 ± 3	42 ± 18	2.7 ± 0.9*	126 [34 - 290]*

Centrilobular	Yes	Idiopathic	4	39 ± 20	9 ± 5	56 ± 16	2.1 ± 0.5	487 [236 - 715]
		
ground-glass		CTD	5	54 ± 24	9 ± 4	49 ± 15	2.0 ± 0.5	148 [141 - 959]
	
attenuation	No	Idiopathic	14	45 ± 19	8 ± 3	61 ± 18	2.3 ± 0.7	263 [46 - 806]
		
		CTD	14	55 ± 13	6 ± 3	45 ± 20	2.7 ± 0.9	111 [34 - 334]

Values are number (percent) or mean ± SD, as appropriate. Data of BNP are shown as median [25percentile, 75percentile]. *P *values for the comparison between the patients with and without each radiographic surrogate for obstruction of the pulmonary veins; *: < 0.02 and **: < 0.01. *P *values for the comparison for the difference between the causes; ^# ^: < 0.03. No mark means that there is no significant effect of either the presence and absence of each radiographic surrogate or the difference in the cause. ^§^: The values for mRAP and mPAP are based on 26 patients. BNP = brain natriuretic peptide, CI = cardiac index, CTD = connective tissue disorder, mRAP = mean right atrial pressure, mPAP = mean pulmonary arterial pressure, RV = right ventricle, SD = standard deviation.

Radiographic surrogates tended to appear with each other. Four patients had three surrogates; nine patients had two surrogates; two patients had one surrogate, and 22 patients had no surrogates.

### Survival rate and radiographic surrogates for obstruction of the pulmonary veins

**Figure 2:**
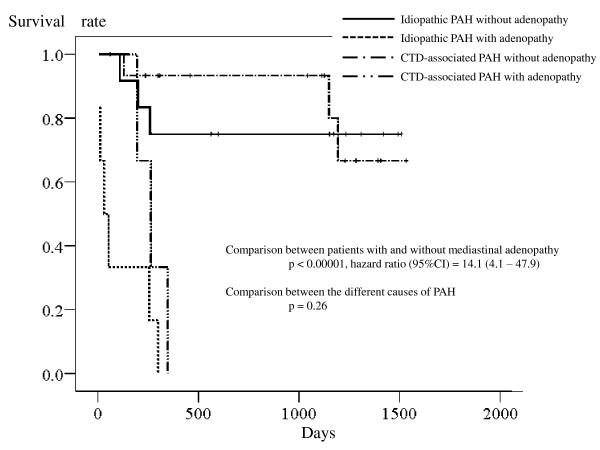
**Kaplan-Meier survival curves of the patients showing the comparison between the presence or lack of mediastinal adenopathy and between idiopathic and connective-tissue-disorder-associated pulmonary arterial hypertension**.

Survival analyses showed that each radiographic surrogate for pulmonary venous obstruction was associated with a seriously increased risk of death. Cox proportional hazard analysis using mediastinal adenopathy as a surrogate found that the adenopathy was associated with a seriously increased risk of death (*p *< 0.0001; hazard ratio [95%CI]) = 13.9 [4.2 - 47.6]). Adding the cause of PAH to the covariate did not weaken the relationship between mediastinal adenopathy and the risk of death (adenopathy: *p *< 0.00001, hazard ratio [95%CI]) = 14.1 [4.1 - 47.9]; causes of PAH: *p *= 0.26); the survival curves of patients with each cause of PAH were close after being stratified by the presence or absence of mediastinal adenopathy (Figure 2). Given the relationship with an elevated plasma concentration of BNP, Cox proportional analysis was also performed by adding the plasma BNP concentration to the covariates. The analysis showed that the adenopathy was associated with a seriously increased mortality rate independently of the BNP concentration (adenopathy: *p *= 0.006, hazard ratio [95%CI] = 7.0 [1.9 - 26.3]; BNP: *p *= 0.013, hazard ratio [95%CI] = 2.0 [1.2 - 3.4]; the causes of PAH: *p *= 0.78).

**Figure 3:**
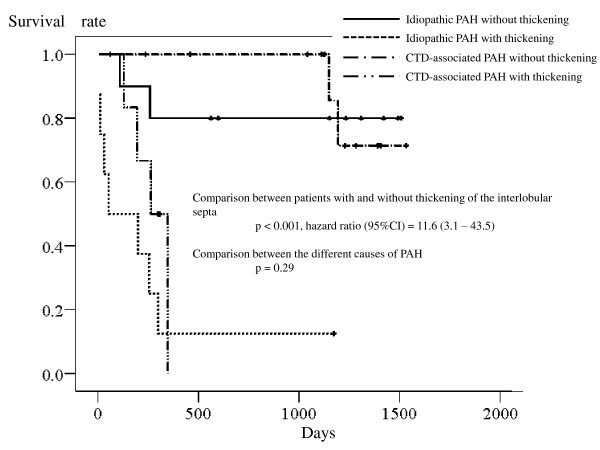
**Kaplan-Meier survival curves of the patients showing the comparison between the presence and lack of thickening of the interlobular septa and between idiopathic and connective-tissue-disorder-associated pulmonary arterial hypertension**.

Cox proportional hazard analysis found that thickening of the interlobular septa was also associated with a seriously increased risk of death (*p *< 0.001; hazard ratio [95%CI] = 12.0 [3.2 - 45.5]). Adding the cause of PAH to the covariate had little impact on the relationship between this surrogate and the risk of death (thickening: *p *< 0.001, hazard ratio [95%CI] = 11.6 [3.1 - 43.5]; the causes of PAH: *p *= 0.29); the survival curves of patients with each cause of PAH were, similar to the results of mediastinal adenopathy, close after stratification by the presence or absence of thickening of the interlobular septa (Figure 3). Given the relationship with an elevated plasma BNP concentration, Cox proportional analysis was also performed by adding the plasma BNP concentration to the covariates. The analysis again showed that interlobular septal thickening was associated with a seriously increased mortality rate independently of the concentration of BNP (thickening: *p *= 0.004, hazard ratio [95%CI] = 8.9 [1.9 - 41.7]; BNP: p = 0.005, hazard ratio [95%CI] = 2.0 [1.3 - 3.3]; the causes of PAH: *p *= 0.30).

**Figure 4:**
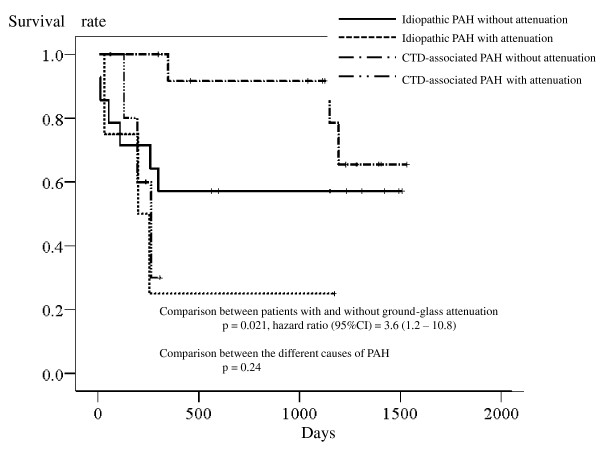
**Kaplan-Meier survival curves of the patients showing the comparison between the presence and lack of ground-glass attenuation and between idiopathic and connective-tissue-disorder-associated pulmonary arterial hypertension**.

The presence of centrilobular ground-glass attenuation was also associated with an increased risk of death, although the effect with this surrogate was somewhat milder than with the other surrogates (*p *= 0.02, hazard ratio [95%CI] = 3.7 [1.2 - 11.1]). Adding the causes of PAH to the covariate had little impact on the relationship between this surrogate and the risk of death (attenuation: *p *= 0.02, hazard ratio [95%CI] = 3.6 [1.2 - 10.8]; the causes of PAH: *p *= 0.24); the survival curves were somewhat close in patients with ground glass attenuation regardless the cause of PAH, although this is less clear than with the other two surrogates (Figure 4). Despite the relationship between this surrogate and the elevation of plasma BNP level did not reach the statistical significance, Cox proportional analysis was performed by adding plasma concentration of BNP to the covariates. The analysis again showed that ground-glass attenuation was associated with a seriously increased mortality rate independently of the BNP concentration (attenuation: *p *= 0.016, hazard ratio [95%CI] = 4.8 [1.3 - 16.9]; BNP: *p *< 0.001; hazard ratio [95%CI] = 2.7 [1.6 - 4.5]; the causes of PAH: *p *= 0.15).

## Discussion

Few studies have assessed obstruction of the pulmonary veins for the risk of death in patients with PAH. The results of the current investigation imply that pulmonary venous obstruction is associated with an elevated risk of death in patients with either idiopathic PAH or CTD-associated PAH. The risk is very large for both causes of PAH. This implication is based on the results of Cox proportional hazard analysis using each radiographic surrogate of pulmonary venous obstruction as follows: mediastinal adenopathy, hazard ratio for death = 13.9 (*p *< 0.0001); thickening of the interlobular septa, hazard ratio = 12.0 (*p *< 0.001); and ground-glass attenuation, hazard ratio = 2.0 (*p *= 0.02).

Recent studies found that obstruction of the pulmonary veins develops in considerable proportion of patients with idiopathic or CTD-associated PAH [3,4]. Dorfmüller et al. report that 75% of patients with CTD-associated and 17% of patients with non-CTD-associated PAH showed obstruction of the pulmonary veins [3]. Overbeek et al. reported that pulmonary venous obstruction was present in 87.5% of patients with scleroderma-associated PAH and in 37.5% of patients with idiopathic PAH [4]. Thus, the differentiation of clinical entities between PAH and PVOD is currently challenged.

For several reasons, obstruction of the pulmonary veins is believed to be related to a malignant prognosis. First, most case reports show that patients with pulmonary venous obstruction die within two years of diagnosis [5-8]; this seems much worse than typical patients with PAH [1,2]. Second, Resten et al. suggest that radiographic surrogates of pulmonary venous obstruction are associated with a poor prognosis [17]. The last report is very important; however, the authors' conclusions were weakened by the study's retrospective design and a lack of survival time analyses [17]. Other than this report, few reports exist examining the effect of pulmonary venous obstruction on the survival of patients with PAH.

In the current study, radiographic surrogates for the pulmonary venous obstruction had a serious impact on the survival of the patients (Figures 2, 3, and 4). In addition, the surrogates were associated with an increased risk of death independently of the baseline BNP plasma concentration. Therefore, the effect of pulmonary venous obstruction on the mortality rate seemed independent of a poor baseline condition. The survival rate of patients with each surrogate is similar to the reported survival rate of patients with proven pulmonary venous obstruction (as assessed by pathological examinations) [5-8]. Thus, the results of the current study suggest great significance of pulmonary venous obstruction as a mortality determinant.

Studies find that the mortality rate of CTD-associated PAH is either double or triple that of idiopathic PAH [22-25]. The reason for this discrepancy, however, is unclear. The results of the current study may provide a suggestion of possible cause of this discordance. As described above, one-half or more of patients with CTD-associated PAH have pulmonary venous obstruction, whilst only a minority of patients with idiopathic PAH do [3,4]. In the present study, dividing the patients by the presence or absence of each radiographic surrogate for pulmonary venous obstruction made the survival curves of patients with each cause of PAH close (Figure 2, 3, and 4). These findings suggest, therefore, that the reason for the generally worse prognosis of CTD-associated PAH, compared with idiopathic PAH, is explained by the difference in the frequency of pulmonary venous obstruction at least in part. Given a lack of statistical significance in the difference of mortality rate between the causes of PAH in this particular patients group, further studies are needed to test this possible explanation for the discordance in prognosis.

Radiographic signs can be used as surrogates as in this study [15]. Studies find that pulmonary radiographic signs on high-resolution CT scan images are successful surrogates for obstruction of the pulmonary veins [15]. A direct diagnosis of pulmonary venous obstruction requires a lung biopsy [11,12]. However, this procedure is hazardous and is available only for select patients [11,12]. In addition, guidelines discourage performing a biopsy in frail PAH patients [11,12]. Thickening of the interlobular septa, mediastinal adenopathy, and ground-glass attenuation are characteristic signs on CT images for obstruction of the pulmonary veins [11,12,15]. A previous study demonstrates that the sensitivity and specificity for pulmonary venous obstruction of each radiographic surrogate are as follows: mediastinal adenopathy, 80% and 100%, respectively; thickening of the interstitial septa, 93%, and 87%, respectively; and the presence of ground-glass attenuation, 87%, and 67%, respectively [15]. Results of two other studies using CT scans agree with these findings [8,26]. The results of pathological studies additionally support the rationale for using radiographic surrogates. Pathological studies demonstrate that interstitial pulmonary edema is observed in a few patients without and most patients with obstruction of the pulmonary veins [3-5,7,8,13,14,26]. Mediastinal adenopathy is moreover recognised as a frequent and very suggestive radiological feature of pulmonary venous obstruction [8,15,16,26]. From these findings, it is apparently possible to use radiographic signs as surrogates for obstruction of the pulmonary veins as in this study. In consideration of the procedural risks, the current study refrained from performing invasive lung biopsies; however, we believe the conclusions of this study are acceptable.

This study had several limitations. This is a single centre investigation; this study therefore has referral biases. We accepted only select patients - the majority of whom had experienced treatment failure at their referring hospitals. This bias was reflected by the poor prognosis of the study patients, the relatively frequent pulmonary venous obstruction among the patients with idiopathic PAH, and a lack of statistical significance in the difference in mortality rate between the patients with idiopathic PAH and those with CTD-associated PAH. Because of the limited number of the patients, the predictive values of each radiographic surrogate were not compared with other characteristics but plasma concentration of BNP. The statistical analyses may have been weak due to the small samples size. Despite of the excellent accuracy, the radiographic surrogates might not completely detect pulmonary venous obstruction.

## Conclusions

In conclusion, the results of this study imply that obstruction of the pulmonary veins is associated with a seriously increased risk of death in patients with PAH.

## List of Abbreviations

BNP: brain natriuretic peptide; CT: computed tomography; CTD: connective tissue disorder; PAH: pulmonary arterial hypertension; PVOD: pulmonary veno-occlusive disease; WHO: World Health Organisation.

## Competing interests

The authors declare that they have no competing interests.

## Authors' contributions

The authors contributed to the study in the following ways: Y. Takeda (Yasuko) participated in the design of the study, obtained the patients' consent, analyzed the reports on CT image, and drafted the manuscript. Yutaka T conceived of the study, participated in its design and coordination, and analyzed the reports on CT image. KY, ST, HN, NO, and GK participated in the design of the study, performed the statistical analysis, and participated to drafting the manuscript. All authors have read and approved the final manuscript.

## Pre-publication history

The pre-publication history for this paper can be accessed here:

http://www.biomedcentral.com/1471-2466/11/47/prepub
